# Perception and reality: The mismatch between absolute and relative physical activity intensity during pregnancy and postpartum in United States women

**DOI:** 10.1016/j.ypmed.2024.107948

**Published:** 2024-05

**Authors:** Kathryn R. Hesketh, Fang Wen, Amy H. Herring, Anna Maria Siega-Riz, Kelly R. Evenson

**Affiliations:** a Department of Epidemiology, Gillings School of Global Public Health, University of North Carolina, Chapel Hill, NC, USA; b MRC Epidemiology Unit, University of Cambridge, Cambridge, UK; c Department of Biostatistics, Gillings School of Global Public Health, University of North Carolina, Chapel Hill, NC, USA; d Department of Statistical Science, Duke University, Durham, NC, USA; e Departments of Nutrition and Biostatistics & Epidemiology, School of Public Health and Health Sciences, University of Massachusetts Amherst, MA, USA

**Keywords:** Physical activity, Pregnancy, Postpartum, Perception, Intensity

## Abstract

**Objective:**

To explore whether a mismatch between absolute physical activity intensity (PAI) and relative self-reported PAI exists during pregnancy and postpartum.

**Methods:**

Women from the PIN3/Postpartum study completed physical activity questionnaires during pregnancy (*n* = 770; Trimester 2: T2, Trimester 3: T3) and postpartum (*n* = 181; 3 months: PP3, 12 months PP12) (2001–2005). Activities women engaged in were assigned Metabolic Equivalent (MET) values for absolute intensity; women self-reported perceived exertion (using the Borg scale) for each activity to provide relative intensity. Hierarchical regression models were used to determine whether a mismatch between absolute and relative PAI (for moderate or vigorous physical activity (MPA; VPA)) differed during pregnancy and postpartum. Models were adjusted for socio-demographic factors.

**Results:**

Women commonly overestimated the amount of MPA and VPA they engaged in [T2 MPA mean 60.5 min/week (49.1, 72.0), VPA 3.7 (−1.4, 8.8); T3: MPA 47.7 (38.9, 56.4), 2.9 (−1.7, 7.4); PP3: MPA 69.5 (43.9, 95.1), VPA 15.8 (1.8, 29.7); PP12: MPA 42.20 (26.8, 57.6), VPA 2.75 (−7.8, 12.9)]. Women overestimated both MPA and VPA to a lesser extent at T3 compared to T2 (MPA: β for difference:-12.6 [95%CI: −26.0, −0.9]; VPA: −0.9 [−6.4, 4.6]). Women continued to overestimate their MPA at PP3 and PP12.

**Conclusions:**

Compared to absolute PAI, perceived PAI was greater for MPA compared to VPA and differences persisted from pregnancy through postpartum. Future research should focus on how perceptions relate to women's actual physiological capacity and whether this mismatch influences the amount of physical activity women engage in during the transition to motherhood.

## Highlights

• Mismatched actual vs perceived physical activity (PA) intensity may impact total PA.
• Pregnant US women overestimated moderate and vigorous intensity PA.
• Women continued to overestimate moderate intensity PA postpartum.
• Mismatches may prevent engagement in sufficient PA and hamper intervention success.

## 1 Introduction

Being physically active provides benefits across the life-course (US Department of Health and Human Services, 2018; Davies et al., 2019), including during pregnancy and the postpartum period (American College of Obstetricians and Gynecologists, 2020). Women without complicated pregnancies are encouraged to engage in regular physical activity to support gestational weight gain management and physical fitness, reduce the risk of gestational diabetes, and boost their mental well-being (American College of Obstetricians and Gynecologists, 2015). International guidance (Bull et al., 2020) recommends that pregnant and postpartum women participate in at least 150 min per week of moderate-intensity aerobic activity; women who are usually active at vigorous intensities can continue to be so. Whilst pre-existing conditions (Meah et al., 2020), changing hormones and physiology (Vannuccini et al., 2022; Farsetti and Valensise, 2022) throughout pregnancy may prevent pregnant women from being physical active, evidence suggest that it is not associated with adverse outcomes such as preterm/prelabour rupture of membranes, fatigue, injury, and musculoskeletal trauma (Davenport et al., 2019). The American College of Obstetrics and Gynecology therefore advocate that women free from contra-indications can be active for 30–60 min on at least 3–4 (but up to 7) days, in thermoneutral or controlled conditions (air conditioning; avoiding prolonged exposure to heat), with supervision if available, until delivery (as tolerated) (American College of Obstetricians and Gynecologists, 2020).

Despite physical activity guidelines, evidence in women without contra-indications suggests that key life transition are associated with decreases in physical activity (Lane-Cordova et al., 2022), with many women in the United States (US) engaging in low levels of physical activity during pregnancy based on their self-report (Evenson and Wen, 2010a; Hesketh and Evenson, 2016). When objective measures are used (e.g., accelerometer compared to self-report), physical activity appears even lower, both during pregnancy and postpartum (Evenson and Wen, 2011; Evenson et al., 2012). Although it has been suggested that, depending on placement, accelerometers may underestimate physical activity during pregnancy (Marshall et al., 2023; Connolly et al., 2011), many women fail to meet physical activity guidelines during pregnancy. This means that if women perceive their physical activity levels to be higher than they actually are, they would plausibly be less likely to increase their physical activity to meet recommended levels. Thus, it is important to determine if and how women's perceived physical activity during pregnancy and postpartum differ from the absolute levels of physical activity undertaken.

Absolute physical activity intensity (PAI) is determined by the actual rate of work (U.S. Department of Health and Human Services, 2008), and for aerobic activity is usually defined as the rate of energy expenditure. This can be described in terms of milliliters per kilograms per minute of oxygen being consumed or Metabolic Equivalent (METs). This is usually assessed by assigning compendium codes (Ainsworth et al., 2011) for which a wide range of physical activities are allocated MET values. Compendium codes classifying absolute PAI during pregnancy do not exist, but codes used in non-pregnant populations are routinely used to estimate likely absolute PAI during pregnancy (Evenson and Wen, 2010b). Absolute PAI can also be assessed using the speed of the physical activity (e.g., walking at 3 miles or jogging at 6 miles per hour), by the physiologic response to intensity (e.g., heart rate), or for resistance activity, expressed as the amount of weight lifted or moved (U.S. Department of Health and Human Services, 2008).

In contrast, perceived PAI depends on a number of individual characteristics, including age and cardiorespiratory fitness, and takes into account a person's physiological capacity (U.S. Department of Health and Human Services, 2008). Relative PAI can therefore be defined physiologically (e.g., percentage of a person's aerobic capacity (VO_2_max) or measured/ estimated maximum heart rate), or expressed as an index of how hard a person feels they are working. This latter subjective assessment of how hard a person is working (relative to capacity) for both aerobic and strengthening activities is called *rating of perceived exertion*, and is frequently assessed using the numerical Borg scale (Borg, 1982), ranging from 6 to 20 and approximates heart rate from 60 to 200 beats/min. The ACOG 2020 guidelines suggest that pregnant women exercise at a medium intensity (12–14 on Borg scale) and at <60–80% of age predicted maximum heart rate, usually not exceeding a heart rate of 140 beats per minute (American College of Obstetricians and Gynecologists, 2020). Thus a perceived rating of exertion may be an alternative way for women to monitor their physical activity intensity, in contrast to more common heart-rate measures (American College of Obstetricians and Gynecologists, 2024). As such, women should engage in physical activity that they rate as “somewhat hard” on the Borg scale of perceived exertion, or when using the “talk test”, be able to carry out a conversation to prevent over-exerting herself.

Limited evidence in non-pregnant populations suggests that adults both under and over-estimate physical activity to differing extents depending on the intensity of that activity and age of participant (Wilcox et al., 2001; Canning et al., 2014). During pregnancy, both absolute PAI and physiological capacity (i.e. relative or perceived PAI) are subject to change (Evenson and Hesketh, 2023). Many physiological changes occur during pregnancy, often moderated by highly complex neuroendocrine mechanisms (Vannuccini et al., 2022); for example, cardiac and renal output and respiration rate all increase, with surges in hormones such as progesterone providing potential benefits to exercise performance (Melzer et al., 2010). However, these changes may result in a mismatch between absolute and perceived PAI during pregnancy, which decreases again during the postpartum period when physiological adaptations wane. Notwithstanding potential measurement error, such a mismatch may lead to conflicting interpretations of energy expenditure and importantly, the related health benefits of any physical activity undertaken. Given women may experience blunted and/or normal heart-rate responses when exercising during pregnancy, it may be difficult for women to determine how hard they are working (U.S. Department of Health and Human Services, 2008). Moreover, if women perceive their physical activity to be higher (or perceive that they are working harder) than they actually are, they may be less inclined to increase their physical activity to meet guideline recommendations.

To our knowledge, this mismatch between absolute and perceived PAI has not been assessed during pregnancy. Establishing whether a mismatch occurs may be beneficial for a number of reasons, including to ensure that healthcare professionals and women themselves are aware of how changing physiology may impact actual and/or perceptions of activity intensity (Evenson and Hesketh, 2023) and to explore ways to better encourage adherence to physical activity guidelines. This study therefore aimed to compare agreement between absolute compendium-coded and perceived PAI during pregnancy and postpartum in a sample of US women. We hypothesized the mismatch would be greater during pregnancy compared to 12 months postpartum and would also be greater for vigorous physical activity compared to moderate intensity physical activity.

## 2 Methods

### 2.1 Participants

Participants were from the Pregnancy, Infection, and Nutrition 3 (PIN3) Study, recruited before 20 weeks' gestation from the University of North Carolina prenatal care clinics (January 2001–June 2005). Women were excluded if they were under 16 years old (with no upper age limit for inclusion), did not speak fluent English, did not plan to receive care or deliver at the study site, had multiple gestations, or did not have access to a telephone to participate in telephone interviews. A total of 1169 women were eligible for the PIN Postpartum Study, an extension of the PIN3 Study for a subset of women delivering between October 2002 and December 2005. PIN3 participants who continued to live in the study area were invited to participate in a home visit at 3-months (*n* = 688) and 12-months (*n* = 550) postpartum for data collection. Details about both the PIN3 and postpartum studies are available elsewhere (Evenson and Wen, 2010b; Siega-Riz et al., 2010); only a woman's first eligible pregnancy could be included in the PIN cohort (i.e. some women had multiple pregnancies during the recruitment period, but only their first during the period was eligible for the study. This didn't have to be their first baby). Women provided informed consent prior to participation. The University of North Carolina Institutional Review Board approved both this analysis and the PIN3 cohort study protocols.

### 2.2 Physical activity intensity derivation

A one-week recall questionnaire was developed for this study (Evenson and Wen, 2010b) to be used repeatedly during pregnancy (17–20 and 27–30 weeks [i.e. T2 and T3]) and postpartum (3 and 12 months postpartum [i.e. PP3 and PP12]). This questionnaire was interviewer-administered and designed to capture all moderate-to-vigorous physical activity (MVPA) in the past week, with evidence of both reliability and validity among pregnant women (Evenson and Wen, 2010b). The questionnaire assessed frequency and duration of all MVPA the woman participated in including activity done at work; for leisure, transportation, childcare, and adult care; and both indoor and outdoor household activities. Women were also asked to rate the perceived (or relative) intensity for each of these activities, with options including fairly light, somewhat hard, hard, or very hard. ‘Fairly light’ activities were excluded to avoid misclassification. The physical activity questionnaire therefore provided an estimate of the total number of minutes in the past week women engaged in moderate and vigorous physical activities, which was used to yield: (i) Absolute PAI using published metabolic equivalent (MET) (Ainsworth et al., 2011), and (ii) Relative PAI based on the modified Borg scale (Borg, 1982). Minutes per week each woman spent in MPA (4.8–7.2 METS) and VPA (>7.2 METS) whilst ‘exercising’ [based on compendium codes for non-pregnant individuals (Ainsworth et al., 2011)] for both absolute and perceived PAI were derived. Analyses were conducted using the above MET classifications for absolute intensity, as these are the bounds recommended for 20 to 39 year olds (Pollock et al., 1998).

### 2.3 Additional variables

Sociodemographic, behavioral, and health related potential confounding factors were explored using Direct Acyclic Diagrams (DAGs). Maternal age at conception, race/ethnicity, educational attainment, parity, perception of general health, and pre-pregnancy body mass index (BMI; derived from self-reported height and weight) were reported at the 17–20 week interview. Women also reported whether physical activity was contraindicated (i.e. they required ‘bedrest’) at 17–20 and 27–30 weeks gestation. Women reported work status (yes/no) at each interview. Women completed the Perceived Stress Scale (short form) (Cohen and Williamson, 1988), shown to have good internal consistency to assess exposure to chronic stress and people's ability to cope (Cohen et al., 1983). Administered as 14 items at T2 and 10 items at subsequent visits, items were scored on a 5-point scale [Never (0) to almost always (4)]. Positively worded items were reverse scored to generate an index indicative of perceived stress where higher values signify higher stresses. To account for differences in the number of questions asked between visits, categorical variables were derived to ensure equivalence across visits (T2: low: 0 ≤ 16; moderate: 17–22; high: ≥23; T3, PP3, PP12: low: 0 ≤ 10; moderate: 11–16; high:≥17).

### 2.4 Statistical analysis

Basic descriptive characteristics of women included in the analysis sample(s) were derived and compared to women in the PIN3 sample who did not provide valid data (i.e. did not have absolute and perceived PAI data at one time point). Median and interquartile ranges for average minutes per week women spent exercising in MPA and VPA for both absolute and perceived PAI were also reported to assess selection bias. Two cohorts were used for analysis: 1) Pregnancy: including all cohort members with complete data at 17–20 and 27–30 weeks for all included variables (*n* = 770), 2) Cohort: all those who had complete data for all four time points (i.e. T2, T3, PP3 and PP12; *n* = 183).

First, using absolute and perceived PAI [i.e. minutes per week each woman spent in MPA and VPA whilst ‘exercising’ (based on compendium codes for non-pregnant individuals (Ainsworth et al., 2011)) for both absolute and perceived PAI], we derived the mean difference between absolute and perceived PAI at each time point [perceived - absolute PAI minutes per week; + value indicates over-estimation, − value indicates under-estimation, 0 indicates no difference]. Then, in order to compare within-person change in absolute vs. perceived PAI across time points, we used hierarchical linear regression (i.e., level 1: time point, level 2: person), including a main effect of time. Analyses were conducted in two stages, adjusting for a) relevant level of baseline physical activity (i.e. level of MPA and VPA at each time point) and b) additional explanatory variables: maternal age at conception (centered to sample mean of 30 years); race (non-Hispanic white, non-Hispanic black, other); level of educational attainment (<13, 13–15, >15 years of education); pre-pregnancy BMI; perceived stress (low, moderate, high); and work status (yes/no). Plots of residual vs. fitted values were inspected to ensure appropriate model fit. All analyses were conducted using STATA 14/SE (College Station, Texas, US), using an alpha of 0.05 to indicate statistical significance.

Only women who provided an estimate of their physical activity level at each time point were included in analyses to ensure that appropriate comparisons could be made between their reported absolute and perceived PAI. We conducted sensitivity analyses to assess whether findings differed when a measure of bedrest (i.e., whether physical activity was contraindicated during pregnancy) was included in the pregnancy sample analysis. Finally, we tested whether a mismatch between absolute and perceived PAI was greater for specific physical activities (i.e. walking; indoor household chores) for women reporting engaging in the same activities at all four time points.

## 3 Results

Descriptive characteristics of the pregnancy and cohort samples are shown in Table 1. Similar socio-demographic differences were also apparent when comparing the pregnancy only (i.e. those providing data at 17–20 and 27–30 weeks (*n* = 770)), and cohort samples (complete data for all four time points (*n* = 183)) (see Table 1). Compared to women participating in the original PIN3 cohort (*n* = 1098), women included in the pregnancy analyses here were significantly older, more likely to be non-Hispanic white, married, more highly educated, work during pregnancy (but not at PP12), and to have a lower pre-pregnancy BMI (*data not shown*).

**Table 1:** Descriptive characteristics of US women participating in the PIN3 (pregnancy sample, *n* = 770) and PIN Postpartum studies (cohort sample, *n* = 183), included in analyses (2001–2005).

	Pregnancy sample n = 770	Cohort sample n = 183
Age in years (mean(SD))	30.0 (5.1)	31.3 (4.6)
**Race/ ethnicity (n(%))** Non Hispanic white Non Hispanic black Other	602 (78.2) 84 (10.9) 84 (10.9)	162 (88.5) 6 (3.3) 15 (8.2)
**Marital status (n(%))** Married Not married	647 (84.0) 123 (16.0)	177 (96.7) 6 (3.3)
**Education in years (n(%))** ≤12 years 13–15 years ≥16 years	98 (12.7) 104 (13.5) 568 (73.8)	5 (2.7) 15 (8.2) 163 (89.1)
**General health (n(%))** Excellent Very good Good Fair/ poor	306 (39.7) 331 (43.0) 110 (14.3) 23 (3.0)	85 (46.5) 79 (43.2) 18 (9.8) 1 (0.6)
**Parity (n(%))** 0 1 2 3+	394 (51.1) 273 (35.5) 80 (10.4) 23 (3.0)	88 (48.1) 76 (41.5) 14 (7.7) 5 (2.7)
**Working status (T3) (n(%))** Employed Not employed	502 (65.2) 268 (34.8)	118 (64.5) 65 (35.5)
Pre-pregnancy BMI (mean(SD))	24.5 (6.2)	25.1 (6.7)
Gestational weight gain (mean(SD))	15.5 (5.5)	15.5 (5.8)
**Bedrest during pregnancy (n(%))** No Yes	742 (96.4) 28 (3.6)	178 (97.3) 5 (2.7)
**Perceived stress (T2) (n(%))** Low Moderate High	302 (39.3) 244 (31.7) 223 (29.0)	79 (43.2) 57 (31.2) 47 (25.7)
**Perceived stress (T3) (n(%))** Low Moderate High	285 (37.1) 292 (38.0) 192 (25.0)	76 (41.5) 75 (41.0) 32 (17.5)
Weight change PP3^⁎^ (kg, mean(SD))		7.9 (9.3)
Weight change PP12^^^ (kg, mean(SD))		3.1 (11.0)
**Working PP3 (n(%))** Unemployed Employed		104 (56.8) 79 (43.2)
**Working PP12 (n(%))** Unemployed Employed		82 (44.8) 101 (55.2)
**Perceived stress PP3 (n(%))** Low Moderate High		79 (43.2) 70 (38.3) 34 (18.6)
**Perceived stress PP12 (n(%))** Low Moderate High		80 (43.7) 64 (35.0) 39 (21.3)

SD: Standard deviation; BMI: Body mass index; T2: Trimester 2; T3: Trimester 3; PP3: 3 months Postpartum; PP12: 12 months Postpartum; * Difference between reported pre-pregnancy and PP3 weight in kg; ^ Difference between reported pre-pregnancy and PP12 weight in kg.

### 3.1 Absolute vs relative physical activity intensity

At each of the four time points (i.e., T2, T3, PP3 and PP12), women over-estimated, on average, the amount of moderate physical activity that they engaged in (≥4.8 METs; Table 2). Data from the cohort sample showed that women also over-estimated VPA during the postpartum period, with the largest mismatch occurring at PP3 for both moderate and vigorous physical activity.

**Table 2:** Mean difference in Absolute vs Relative Physical Activity Intensity (PAI) across pregnancy (n = 770) and postpartum (n = 183) in a sample of US women.

	Mean difference Minutes per week (95% CI)	
	MPA^a^	VPA^a^
Pregnancy sample (*n* = 770)		
T2	60.5 (49.1, 72.0)	3.7 (−1.4, 8.8)
T3	47.7 (38.9, 56.4)	2.9 (−1.7, 7.4)
Cohort sample (n = 183)		
T2	63.9 (34.5, 93.3)	6.8 (0.8, 14.4)
T3	43.7 (24.1, 63.3)	1.0 (−8.2, 6.1)
PP3	69.5 (43.9, 95.1)	15.8 (1.8, 29.7)
PP12	42.2 (26.8, 57.6)	2.8 (−7.8, 12.9)

a Derived using relative vs. absolute PAI based on activity MET value; positive values signify overestimate at given intensity [e.g. *a* *=* *1, R* *=* *2: R-A* *=* *1*] and negative values signify underestimate at given intensity. T2: Trimester 2; T3: Trimester 3; PP3: 3 months postpartum; PP12: 12 months postpartum; MPA, moderate physical activity; VPA, vigorous physical activity; CI, confidence interval; pregnancy sample: Women who had complete data at both T2 and T3 for all included variables; cohort sample: Women who had complete data for all four time points.

### 3.2 Absolute vs relative physical activity intensity over time

Using hierarchical regression analyses, we assessed the within-person change in the mismatch between absolute and perceived PAI over time. In the pregnancy sample, analyses indicated that women were likely to overestimate the amount of MVPA they engaged in at T2 (MPA: 55.8 [10.1, 101.5]; VPA: 21.3 [1.1, 43.6]), but overestimated their MPA to a lesser extent at T3 (MPA: 43.4 [−2.4, 89.3], VPA: 20.4 [−2.1, 42.8]) (Table 3, Supplementary Table 1).

**Table 3:** Analysis of absolute vs relative physical activity intensity (PAI) across pregnancy (n = 770) and postpartum (n = 183) in a sample of US women.

	Unadjusted Difference β minutes/week [95%CI]		Adjusted⁎ Difference β minutes/week [95%CI]	
	[A-R] MPA	[A-R] VPA	[A-R] MPA	[A-R] VPA
**Pregnancy sample** **(n** **=** **770)**				
T2	67.3 [56.8,77.7]	10.3 [5.5, 15.2]	55.8 [10.1, 101.5]	21.3 [1.1, 43.6]
T3	54.7 [44.2,65.3]	9.41 [4.6, 14.2]	43.4 [−2.4, 89.3]	20.4 [−2.1, 42.8]
**Cohort sample** **(n** **=** **183)**				
T2	70.8 [47.6, 94.0]	−2.9 [−13.2, 7.5]	85.2 [−18.1, 188.5]	−3.8 [−55.4,47.9]
T3	50.6 [27.4, 73.8]	2.9 [−7.4, 13.3]	65.7 [−37.46, 168.9]	2.4 [−49.2, 54.0]
PP3	76.4 [53.2, 99.6]	19.7 [9.4, 30.1]	90.7 [−13.2, 194.6]	−3.8 [−55.4, 47.9]
PP12	49.1 [25.9,72.3]	6.7 [−3.6, 17.1]	63.5 [−40.0, 166.9]	5.0 [−46.7, 56.7]

⁎ Analyses adjusted for maternal age at conception (centred to sample mean of 30 years); race (non-hispanic white, non-hispanic black, other); level of educational attainment (<13, 13–15, >15 years of education); pre-pregnancy BMI; perceived stress (low, moderate, high); and work status (yes/no); T2: Trimester 2; T3: Trimester 3; PP3: 3 months postpartum; PP12: 12 months postpartum; MPA, moderate physical activity; VPA, vigorous physical activity; CI, confidence interval; pregnancy sample: Women who had complete data at both T2 and T3 for all included variables; cohort sample: Women who had complete data for all four time points.

In the cohort sample, the same pattern as the pregnancy sample was apparent for overestimation of MPA at T2 and T3, with higher magnitudes of overestimation in the cohort sample (T2: 85.2 [−18.1, 188.5], T3: 65.7 [−37.46, 168.9]). Women continued to overestimate their MPA at PP3 and PP12, showing overestimation of ∼90 min at PP3 (90.7 [−13.2, 194.6]). In contrast, for VPA, the mismatch was smaller at all time points, with a maximum difference of 5 min observed at PP12 between absolute and relative PAI (5.0 [−46.7, 56.7]).

Findings did not differ when a measure of bedrest (i.e., whether physical activity was contraindicated during pregnancy) was included in the pregnancy sample analysis, and the mismatch between absolute and perceived PAI did not differ for specific physical activities (i.e. walking; indoor household chores) over time.

## 4 Discussion

Across pregnancy and postpartum, we identified a mismatch between women's absolute and perceived PAI. During mid-pregnancy, women tended to over-estimate the amount of MPA and VPA that they engaged. Put another way, on average, for women perceived they were engaging in activities at moderate-to-vigorous intensity, when they were in fact engaging in physical activities classified at a lighter intensity according to the MET values. Assessing individual-level change over time, analyses also suggest that the mismatch between absolute and perceived PAI differed across pregnancy and the postpartum period. The mismatch appeared to be greatest in T2 in both pregnancy and cohort samples, and at PP3 for women providing data from the postpartum period (i.e., the cohort sample). Contrary to our hypothesis, the mismatch was greater for MPA (vs. VPA) which may reflect the fact that women engaged in lower levels of VPA in this sample overall. Further research is required to determine how perceptions relate to women's actual physiological capacity during the transition to motherhood to determine whether these mismatches influence the amount of physical activity women engage in and their rate of work during this period.

Previous evidence in a sample of women (aged 40–91 years) (Wilcox et al., 2001), suggested estimates of perceived PAI were higher for light and vigorous physical activity, and lower for moderate physical activity compared to absolute compendium-derived PAI. Another study assessing the intensity of activity required to obtain health benefits, found that participants underestimated moderate and vigorous effort activity (Canning et al., 2014). Contrastingly, we identified a trend in women over-estimating their moderate-to-vigorous physical activity during pregnancy and into the postpartum period for MPA. This indicates that a woman's perception of how hard she is working does appear to be altered during the transition to motherhood and may continue post birth.

During pregnancy, a wide range of physiological adaptations occur, including changes to the cardiovascular system, metabolism, and respiratory capacity (Soma-Pillay et al., 2016). Many begin during early pregnancy, but change will occur according to physiological need as the fetus grows. Awareness and perception of these physiological changes will also develop over time and may depend on the activities that women engage in. For example, those who are largely sedentary may be less likely to notice physiologic changes compared to those who are active; those who engage in higher levels of MPA may continue to believe they are working as hard during pregnancy, though overall levels of activity may have declined. It is possible that adapting to and perceiving differences in capacity as a result of these changes may therefore stabilize by the third trimester, with our finding suggesting that this may well be the case. However, subsequent reversal of these physiological changes is likely to then require further modulation of women's perceptions during the postpartum period, as is also born out in our findings. Future work to determine how women's perceptions of capacity are related to physiological changes, during pregnancy and postpartum, would be beneficial to delineate whether, and at which stage, changes occur.

A mismatch between absolute and relative PAI has clear implications for measurement and analysis of physical activity, particularly among pregnant and postpartum women. The Borg scale has been suggested as one way in which pregnant women can monitor their own exercise, working at a relative intensity of 13–14 (classified as ‘somewhat hard’) (American College of Obstetricians and Gynecologists, 2024). The training zone according to Borg for healthy adults is 12–16 (Melzer et al., 2010). Whether this continues to be appropriate during pregnancy may be activity specific, with differences seen between weight-supported exercise (e.g. cycling) (Pivarnik et al., 1991) compared to weight-bearing exercise (e.g. walking), as energy expenditure increases or as women gain weight (U.S. Department of Health and Human Services, 2008). Ratings of perceived exertion may also not correlate with exercise heart rates during pregnancy; they tend to underestimated exercise heart-rate in the second trimester during walking, aerobics classes and circuit training, and during cycling and aerobics classes in the third trimester (O'Neill et al., 1992). It is also plausible that women over-estimated their absolute PAI, which would result in smaller mismatches between absolute and relative PAI than presented here. Relatedly, compendium codes used to allocate MET values here are those used for the general adult population (Ainsworth et al., 2011). It is not currently known whether this is appropriate for pregnant and postpartum women, and group-specific compendium codes may be preferable to ensure MET values accurately how hard women are working during the transition to motherhood. Such compendium codes would likely need to be devised using MET values for women at various stages of pregnancy, to account for physiological adaptations across trimesters. The use of device-based measures of physical activity (Evenson et al., 2015) may allow women to better track the frequency, duration, and intensity of their daily physical activity during pregnancy and postpartum to better understand activity accrued and monitor their weekly levels.

Importantly, the number and variation in physical activities that women engaged in were consistent across the measurement period. Walking, home-based chores and playing/ caring for children were the most reported; the latter also increased during the postpartum period (Borodulin et al., 2009). This implies that women were consistently engaging in the same activities, but perceptions of their intensity differed over time. It is probable that women amend their activity behaviours due to changing physiological demands, and/or as a result of social pressure to ‘take it easy’, given long-standing societal perceptions that physical activity is inappropriate during pregnancy (Findley et al., 2020). It should also be noted that women included in analyses here were those who reported engaging in some form of activity during the measurement period. Given many women report engaging in very little physical activity during pregnancy (Evenson and Wen, 2010a; Hesketh and Evenson, 2016), women here present a specific subsample of the pregnant population. We can only hypothesize how those women who are largely sedentary would perceive their physical activity intensity, though it is plausible that they would exhibit an even greater mismatch between absolute and perceived activity intensity. It remains to be determined whether having a greater mismatch between absolute and perceived activity intensity makes a clinical difference in terms of women's health, and whether it matters that a mis-match occurs if women are engaging in some physical activity. However, guidelines suggest that all women, free from medical complications, should engage in physical activity of a moderate intensity throughout the week, for >20–30 min per day (American College of Obstetricians and Gynecologists, 2024). As few women here, and at the population level (Hesketh and Evenson, 2016), meet these guidelines, it is important to ensure that women are aware of the duration, frequency *and* intensity of physical activity required to achieve the related health benefits for themselves and their baby.

### 4.1 Strengths and limitations

Several limitations of this work should be acknowledged. To derive absolute PAI, women were asked to report the average amount of physical activity they engaged in across a range of domains over the past week, that “caused at least some increase in breathing and heart rate”. Based on this, we would expect that at least moderate activity would be reported, and classified women's relative physical activity intensity to those classified as ‘somewhat hard, hard, or very hard’. We chose to exclude “fairly light” activities from analyses here, but a degree of misclassification cannot be ruled out. In addition, women's absolute levels of PAI were derived from compendium codes: during pregnancy, these codes may not equate to women's actual PAI intensity given the physiological changes that occur, but it is likely that any misclassification would be similar across all women. There is a possibility, due to the number of tests conducted, that significant findings may have occurred by chance. Finally, due to factors such as attrition and subsequent pregnancy between 3- and 12-months postpartum, the women included in this study were not representative of the original pregnancy cohort, nor of women more generally, given participants were volunteers from central North Carolina. Confirmation of these findings in population-based cohorts of pregnancy and postpartum women would therefore be beneficial.

## 5 Conclusions

We identified a mismatch between women's absolute and perceived physical activity intensity during both pregnancy and postpartum. During pregnancy women tend to perceive that they were engaging in activities at moderate-vigorous intensity, despite these activities being classified as light intensity according to compendium codes. This trend persists into the postpartum period but starts to normalize one year after birth. Further research is now required to determine how perceptions relate to women's actual physiological capacity; whether this mismatch influences the amount of physical activity women engage in; and if this results in a clinically meaningful difference in the health benefits obtained during the transition to motherhood.

## Funding statement

Funding for this study was provided by the 10.13039/100000002National Institutes of Health (NIH) / 10.13039/100000054National Cancer Institute (#CA109804-01). Data collection was supported by NIH / 10.13039/100000071National Institute of Child Health and Human Development (#HD37584), NIH General Clinical Research Center #RR00046), and NIH / 10.13039/100000062National Institute of Diabetes and Digestive and Kidney Diseases (#DK 061981-02). KH was supported by the 10.13039/100010269Wellcome Trust (107337/Z/15/Z) and the 10.13039/501100000265Medical Research Council [Unit Programme number MC_UU_00006/5]. The content is solely the responsibility of the authors and does not necessarily represent the official views of the funding agencies. The Pregnancy, Infection, and Nutrition Study is a joint effort of many investigators and staff members whose work is gratefully acknowledged. For the purpose of Open Access, the author has applied a Creative Commons Attribution (CC BY) license to any Author Accepted Manuscript version arising from this submission.

## CRediT authorship contribution statement

**Kathryn R. Hesketh:** Writing – review & editing, Writing – original draft, Formal analysis, Data curation, Conceptualization. **Fang Wen:** Writing – review & editing, Writing – original draft, Methodology, Data curation. **Amy H. Herring:** Writing – review & editing, Writing – original draft, Methodology, Data curation. **Anna Maria Siega-Riz:** Writing – review & editing, Writing – original draft, Methodology, Investigation, Funding acquisition. **Kelly R. Evenson:** Writing – review & editing, Writing – original draft, Methodology, Investigation, Funding acquisition, Conceptualization.

## Declaration of competing interest

The authors declare no conflicts of interest.

## Data Availability

Data may be made available on request, as it is stored on a secure server in the USA.

## Appendix A Supplementary data

Supplementary material Image 1

## References

[bb0005] Ainsworth B.E., Haskell W.L., Herrmann S.D. (2011). 2011 compendium of physical activities: a second update of codes and MET values. Med. Sci. Sports Exerc..

[bb0010] American College of Obstetricians and Gynecologists (2015). Physical activity and exercise during pregnancy and the postpartum period. Committee opinion No. 650. Obstet. Gynecol..

[bb0015] American College of Obstetricians and Gynecologists (2020). Committee on obstetric practice physical activity and exercise during pregnancy and the postpartum period. Obstet. Gynecol..

[bb0020] American College of Obstetricians and Gynecologists (2024).

[bb0025] Borg G. (1982). Psychophysical bases of perceived exertion. Med. Sci. Sports Exerc..

[bb0030] Borodulin K., Evenson K.R., Herring A.H. (2009). Physical activity patterns during pregnancy through postpartum. BMC Womens Health.

[bb0035] Bull F.C., Al-Ansari S.S., Biddle S. (2020). World Health Organization 2020 guidelines on physical activity and sedentary behaviour. Br. J. Sports Med..

[bb0040] Canning K.L., Brown R.E., Jamnik V.K., Salmon A., Ardern C.I., Kuk J.L. (2014). Individuals underestimate moderate and vigorous intensity physical activity. PloS One.

[bb0045] Cohen S., Williamson G. (1988).

[bb0050] Cohen S., Kamarck T., Mermelstein R. (1983). A global measure of perceived stress. J. Health Soc. Behav..

[bb0055] Connolly C.P., Coe D.P., Kendrick J.M., Bassett D.R., Thompson D.L. (2011). Accuracy of physical activity monitors in pregnant women. Med. Sci. Sports Exerc..

[bb0060] Davenport M.H., Ruchat S.M., Sobierajski F. (2019). Impact of prenatal exercise on maternal harms, labour and delivery outcomes: a systematic review and meta-analysis. Br. J. Sports Med..

[bb0065] Davies D.S.C., Atherton F., McBride M., Calderwood C. (2019).

[bb0070] Evenson K.R., Hesketh K.R. (2023). Monitoring physical activity intensity during pregnancy. Am. J. Lifestyle Med..

[bb0075] Evenson K.R., Wen F. (2010). National trends in self-reported physical activity and sedentary behaviors among pregnant women: NHANES 1999-2006. Prev Med (Baltim).

[bb0080] Evenson K.R., Wen F. (2010). Measuring physical activity among pregnant women using a structured one-week recall questionnaire: evidence for validity and reliability. Int. J. Behav. Nutr. Phys. Act..

[bb0085] Evenson K.R., Wen F. (2011). Prevalence and correlates of objectively measured physical activity and sedentary behavior among US pregnant women. Prev Med (Baltim).

[bb0090] Evenson K.R., Herring A.H., Wen F. (2012). Self-reported and objectively measured physical activity among a cohort of postpartum women: the PIN postpartum study. J. Phys. Act. Health.

[bb0095] Evenson K.R., Goto M.M., Furberg R.D. (2015). Systematic review of the validity and reliability of consumer-wearable activity trackers. Int. J. Behav. Nutr. Phys. Act..

[bb0100] Farsetti D., Valensise H. (2022). Hormones and Pregnancy.

[bb0105] Findley A., Smith D.M., Hesketh K., Keyworth C. (2020). Exploring womens’ experiences and decision making about physical activity during pregnancy and following birth: a qualitative study. BMC Pregnancy Childbirth.

[bb0110] Hesketh K.R., Evenson K.R. (2016). Prevalence of U.S. pregnant women meeting 2015 ACOG physical activity guidelines. Am. J. Prev. Med..

[bb0115] Lane-Cordova A.D., Jerome G.J., Paluch A.E. (2022). Supporting physical activity in patients and populations during life events and transitions: a scientific statement from the American Heart Association. Circulation.

[bb0120] Marshall M.R., Montoye A.H.K., Conway M.R., Schlaff R.A., Pfeiffer K.A., Pivarnik J.M. (2023). Location, location, location: accelerometer placement affects steps-based physical activity outcomes during pregnancy and postpartum. Am. J. Lifestyle Med..

[bb0125] Meah V.L., Davies G.A., Davenport M.H. (2020). Why can’t I exercise during pregnancy? Time to revisit medical ‘absolute’ and ‘relative’ contraindications: systematic review of evidence of harm and a call to action. Br. J. Sports Med..

[bb0130] Melzer K., Schutz Y., Boulvain M., Kayser B. (2010). Physical activity and pregnancy. Sports Med..

[bb0135] O’Neill M.E., Cooper K.A., Mills C.M., Boyce E.S., Hunyor S.N. (1992). Accuracy of Borg’s ratings of perceived exertion in the prediction of heart rates during pregnancy. Br. J. Sports Med..

[bb0140] Pivarnik J., Lee W., Miller J. (1991). Physiological and perceptual responses to cycle and treadmill exercise during pregnancy. Med. Sci. Sports Exerc..

[bb0145] Pollock M., Gaesser G., Butcher J. (1998). American College of Sports Medicine position stand: the recommended quantity and quality of exercise for developing and maintaining fitness in healthy adults. Med. Sci. Sports Exerc..

[bb0150] Siega-Riz A.M., Herring A.H., Carrier K., Evenson K.R., Dole N., Deierlein A. (2010). Sociodemographic, perinatal, behavioral, and psychosocial predictors of weight retention at 3 and 12 months postpartum. Obesity.

[bb0155] Soma-Pillay P., Nelson-Piercy C., Tolppanen H., Mebazaa A. (2016). Physiological changes in pregnancy. Cardiovasc. J. Afr..

[bb0160] U.S. Department of Health and Human Services (2008).

[bb0165] US Department of Health and Human Services (2018). https://health.gov/paguidelines/second-edition/report.aspx.

[bb0170] Vannuccini S., Paternò I., Challis J.R.G., Petraglia F. (2022). Hormones and Pregnancy.

[bb0175] Wilcox S., Irwin M.L., Addy C. (2001). Agreement between participant-rated and compendium-coded intensity of daily activities in a triethnic sample of women ages 40 years and older. Ann. Behav. Med..

