# Supplementary material for: Perception and reality: The mismatch between absolute and relative physical activity intensity during pregnancy and postpartum in United States women
Source: Prev Med. 2024 May;182:107948. doi: 10.1016/j.ypmed.2024.107948 (PMC11079917; doi:10.1016/j.ypmed.2024.107948)
Supplement: Supplementary file 1 — Supplementary material [file mmc1.docx]

# Supplementary Table 1 Comparison of Absolute vs Relative Physical Activity Intensity (PAI) across pregnancy (n=770) and postpartum (n=183) in a sample of US women

|  | Unadjusted  Difference ß minutes/week [95% C.I.] | | Adjusted*  Difference ß minutes/week [95% C.I.] | |
| --- | --- | --- | --- | --- |
| **Pregnancy Sample** | **[A-R] MPA** | **[A-R] VPA** | **[A-R] MPA** | **[A-R] VPA** |
| Baseline (T2) | 67.3 [56.8, 77.7] | 10.3 [5.5, 15.2] | 55.8 [10.1, 101.5] | 21.3 [1.1, 43.6] |
|  |  |  |  |  |
| T3 | -12.6 [-26.0, 2.9] | 0.92 [-6.4, 4.6] | -12.35 [-1.1,25.8] | 0.90 [-4.6,6.4] |
|  |  |  |  |  |
|  |  |  |  |  |
| **Cohort Sample** |  |  |  |  |
| Baseline (T2) | 70.8 [47.6, 94.0] | 2.9 [-13.2, 7.5] | 85.2 [-18.1, 188.5] | -3.8 [-55.4,47.9] |
|  |  |  |  |  |
| T3 | -20.2 [-51.6, 11.3] | 5.79 [-18.75,7.17] | -19.5 [-51.0, 12.2] | 6.2 [-6.8, 19.2] |
|  |  |  |  |  |
| PP3 | 5.6 [-25.8, 37.1] | 22.6 [9.6, 35.5] | 5.5 [-26.4, 37.4] | 20.6 [7.5, 33.8] |
|  |  |  |  |  |
| PP12 | -21.7 [-53.1, 9.8] | 9.6 [-3.4, 22.5] | -21.7 [-53.2, 9.8] | 8.8 [-4.2, 21.7] |
|  |  |  |  |  |
|  |  |  |  |  |
| Baseline (T3) | 50.6 [27.4, 73.8] | 2.9 [-7.4, 13.3] | 65.7 [-37.5,168.9] | 2.4 [-49.1, 54.0] |
|  |  |  |  |  |
| T2 | 20.2 [11.3, 51.6] | -5.8 [-18.8, 7.2] | 19.5 [-12.1, 51.0] | -6.2 [-19.2, 6.8] |
|  |  |  |  |  |
| PP3 | 25.8 [5.7, 57.3] | 16.8 [3.8, 29.8] | 25.0[-6.8, 56.8] | 14.4 [-1.3, 27.6] |
|  |  |  |  |  |
| PP12 | -1.5 [-33.0, 30.0] | 3.8[-9.2, 16.8] | -2.2 [-33.8, 29.3] | 2.6 [-10.4, 15.5] |
|  |  |  |  |  |
| Baseline (PP3) | 76.4 [53.2, 99.6] | 19.7 [9.4, 30.1] | 90.7 [-13.2, 194.6] | -3.8 [-55.4, 47.9] |
|  |  |  |  |  |
| T2 | -5.6 [-37.1, 25.8] | -22.6 [-35.6, -9.6] | -5.5 [-26.4,37.4] | 6.2 [-6.8, 19.2] |
|  |  |  |  |  |
| T3 | -25.8 [-57.3, 5.7] | -16.8 [-29.8, -3.84] | -25.0 [-56.8, 6.8] | 20.6 [7.5, 33.8] |
|  |  |  |  |  |
| PP3 | -27.3 [-58.8, 4.2] | -13.0 [-26.0, -0.05] | -27.2 [-58.8, 4.3] | 8.8 [-4.2, 21.7] |
|  |  |  |  |  |
| Baseline (PP12) | 49.1 [25.9, 72.3] | 6.7 [-3.6, 17.1] | 63.5 [-40.0, 166.9] | 5.0 [-46.7, 56.7] |
|  |  |  |  |  |
| T2 | 21.7 [9.8, 53.1] | -9.6 [-22.5, 3.4] | 21.7 [-9.8, 53.2] | -8.8 [-21.7, 4.2] |
|  |  |  |  |  |
| T3 | 1.5 [-30.0, 33.0] | -3.8 [-16.8, 9.2] | 2.2 [-29.3, 33.8] | -2.6 [-15.5, 10.4] |
|  |  |  |  |  |
| PP3 | 27.3 [4.2, 58.8] | 13.0 [0.05, 26.0] | 27.2 [-4.3, 58.8] | 11.9 [-1.1, 24.9] |
|  |  |  |  |  |

^* Analyses adjusted for maternal age at conception (centred to sample mean of 30 years); race (non-hispanic white, non-hispanic black, other); level of educational attainment (<13, 13-15, >15 years of education); pre-pregnancy BMI; perceived stress (low, moderate, high); and work status (yes/no); T2: Trimester 2; T3: Trimester 3; PP3: 3 months Postpartum; PP12: 12 months Postpartum; Pregnancy sample: women who had complete data at both T2 and T3 for all included variables; Cohort sample: women who had complete data for all four time points.^
